# The New Therapeutic Approaches in the Treatment of Non-Alcoholic Fatty Liver Disease

**DOI:** 10.3390/ijms222413219

**Published:** 2021-12-08

**Authors:** Branka Filipovic, Snezana Lukic, Dragana Mijac, Marija Marjanovic-Haljilji, Marko Vojnovic, Jelena Bogdanovic, Tijana Glisic, Natasa Filipovic, Jamal Al Kiswani, Aleksandra Djokovic, Suncica Kapor, Slobodan Kapor, Zoran Bukumiric, Ana Starcevic

**Affiliations:** 1 Faculty of Medicine, University of Belgrade, Dr Subotica Starijeg 8, 11000 Belgrade, Serbia; lukic.snezana@gmail.com (S.L.); draganamijac@gmail.com (D.M.); jeca.bogdanovic@yahoo.com (J.B.); tijana.glisic78@gmail.com (T.G.); drsaska@yahoo.com (A.D.); kaporbg@gmail.com (S.K.); zoran.bukumiric@med.bg.ac.rs (Z.B.); ana.starcevic22@gmail.com (A.S.); 2 Department of Gastroenterology, Clinical and Hospital Center “Dr Dragisa Misovic-Dedinje”, Heroja Milana Tepica 1, 11020 Belgrade, Serbia; maja-s-92@hotmail.com (M.M.-H.); filipovicn@live.com (N.F.); jamalkiswani@gmail.com (J.A.K.); 3 Clinic of Gastroenterology and Hepatology, Clinical Center of Serbia, Koste Todorovica 2, 11000 Belgrade, Serbia; marko.vojna@gmail.com; 4 Clinic of Endocrinology, Diabetes and Metabolic Disease, Clinical Center of Serbia, Dr Subotica 13, 11000 Belgrade, Serbia; 5 Department of Cardiology, Clinical and Hospital Center “Bezanijska Kosa”, Dr Zorza Matea s/n, 11080 Belgrade, Serbia; 6 Department of Hematology, Clinical and Hospital Center “Dr Dragisa Misovic-Dedinje”, Heroja Milana Tepica 1, 11020 Belgrade, Serbia; suncicabjelica@gmail.com; 7 Institute of Anatomy “Niko Miljanic”, Dr Subotica Starijeg 4/2, 11000 Belgrade, Serbia; 8 Institute of Medical Statistics and Informatics, Faculty of Medicine, University of Belgrade, 11000 Belgrade, Serbia

**Keywords:** non-alcoholic fatty liver disease, lipotoxicity, organelle dysfunction, dysbiosis, new therapeutic modalities

## Abstract

Non-alcoholic fatty liver disease (NAFLD) is the most prevalent chronic liver disease which is characterized by extremely complex pathogenetic mechanisms and multifactorial etiology. Some of the many pathophysiological mechanisms involved in the development of NAFLD include oxidative stress, impaired mitochondrial metabolism, inflammation, gut microbiota, and interaction between the brain-liver-axis and the regulation of hepatic lipid metabolism. The new therapeutic approaches in the treatment of NAFLD are targeting some of these milestones along the pathophysiological pathway and include drugs like agonists of peroxisome proliferator-activated receptors (PPARs), glucagon-like peptide-1 (GLP-1) agonists, sodium/glucose transport protein 2 (SGLT2) inhibitors, farnesoid X receptor (FXR) agonists, probiotics, and symbiotics. Further efforts in biomedical sciences should focus on the investigation of the relationship between the microbiome, liver metabolism, and response to inflammation, systemic consequences of metabolic syndrome.

## 1. Introduction

Nonalcoholic fatty liver disease (NAFLD) is one of the most common causes of chronic liver disease with a prevalence of approximately 30% worldwide [1]. NAFLD is defined as hepatic fat accumulation in >5% of hepatocytes according to histology and can be presented with a spectrum of disease presentations from non-alcoholic fatty liver (NAFL), non-alcoholic steatohepatitis (NASH), fibrotic NASH, to cirrhosis and HCC [2].

About 20% of cases develop non-alcoholic steatohepatitis (NASH), which is a chronic hepatic inflammation that can progress to fibrosis and cirrhosis in approximately 10–20% of patients. NAFLD with advanced fibrosis is the third most common cause of hepatocellular carcinoma (HCC), while HCC may occur in patients with NAFLD even in the absence of cirrhosis. NAFLD currently represents the third major cause of liver transplantation (LT) [3].

NAFLD may be referred to as the hepatic manifestation of the metabolic syndrome (MetS) and a spectrum of metabolic dysfunctions including type 2 diabetes mellitus (T2DM), dyslipidemia, insulin resistance (IR), hypertriglyceridemia, and obesity. The prevalence of NAFLD is 90% in patients with dyslipidemia and 70% among diabetics. Of the adults with NAFLD and T2DM who undergo a liver biopsy, 17% have advanced fibrosis [4]. Sedentary lifestyle and Western diet are the two major risk factors that influence centripetal obesity and IR, and the development of NAFLD [5]. Recently, a new definition was proposed for NAFLD, the metabolic dysfunction-associated fatty liver disease (MAFLD) [6]. The prevalence of MAFLD approaches approximately 50% among obese adults and is higher in men than in women [7]. However, NAFLD is present in 7% of non-obese individuals (lean NAFLD) [8]. An emerging body of evidence suggests that NAFLD is a multisystemic disease, independent of other metabolic comorbidities, associated with cardiovascular disease (CVD), chronic kidney disease (CKD), T2DM, and reduced mineral density. Adults with NAFLD have increased overall mortality, while the most common cause of death in patients with NAFLD is cardiovascular disease (CVD) [9]. Among the top three causes of death in adults with NAFLD is cancer-related mortality.

The mechanisms underlying NAFLD pathogenesis are still poorly understood. It is well known that NAFLD has strong hereditable traits and several genetic polymorphisms on the Patatin-like Phospholipase Domain-containing 3 (*PNPLA3*) genes and Transmembrane 6 Superfamily Member 2 (*TM6SF2*) gene have been identified with associated risks for NAFLD and increased risk for HCC [10].

In this review, we discuss the underlying molecular mechanisms of the pathogenesis of NAFLD, NASH, and their progression to cirrhosis. Following recent investigations in new therapeutic agencies in pre-clinical and clinical trials, potential treatment options will be discussed.

## 2. Etiopathogenesis of NAFLD

### 2.1. General Hypothesis

The etiopathogenetic mechanisms underlying the development of NAFLD and its progression to NASH are rather complex, and despite all the scientific efforts in the last decade are not yet fully understood. Once proposed two-hit theory was replaced by themultiple-hithypothesis, a much more complex explanation, suggesting that there aren’t one or two mechanisms responsible for NAFLD pathogenesis or development of NASH, but more likely a cobweb of simultaneously acting factors that add up to this equation [11].

The main feature of liver steatosis is excessive fat accumulation in the hepatocytes which occurs when the liver capacity to balance between lipid acquisition and discharge has been overcome. Hepatic lipid influx in form of free fatty acids (FFAs) derives mostly from two major sources-lipolysis of triglycerides (TG) in adipose tissue or FFAs synthesis from glucose and fructose by de novo lipogenesis (DNL). The removal of lipid occurs through the processes of mitochondrial fatty acid oxidation (FAO) or the production of very low-density lipoproteins (VLDL). If an impairment occurs somewhere along the way, it can lead to the production of toxic lipid metabolites, which further induce hepatocellular stress—a phenomenon better known as lipotoxicity, the first step of the pathway leading from simple steatosis to NASH [12].

Multiple parallel hypotheses promote several entities that all together contribute to the development of NAFLD, such as dietary factors, obesity, insulin resistance (IR), genetic and epigenetic determinants as well as gut dysbiosis [11]. A pivotal role in this theory may belong to IR, which dysregulates lipolysis in adipose tissue, thus increasing the flux of FFAs to the liver. On the other hand, it is now well known that in obesity adipose tissue dysfunction eventually occurs, which also leads to an increased FFAs liver uptake, inducing increased lipid synthesis and gluconeogenesis, thus promoting hepatic IR even more [13]. Hyperinsulinemia that is present as an attempt to overcome hepatic IR stimulates DNL. DNL can be increased by activation of transcription factors such as sterol regulatory element-binding protein-1 (SREBP-1), carbohydrate response element-binding protein (ChREBP), and peroxisome proliferator-activated receptor (PPAR)-γ [14]. Increased liver fat also promotes hepatic glucagon resistance (GR) over the amino acids (AAs) metabolism reducing urea genesis and resulting in hyperaminoacidemia. Increased AAs stimulate glucagon production to compensate for hepatic GR, and a vicious cycle is installed (the liver-pancreas axis), which leads to hyperglucagonemia and increased hepatic glucose release [15]. Under these hyperglycemic conditions, advanced glycation end-products (AGEs) are generated, most of them toxic, accumulating, and causing cumulative metabolic burden, leading to inflammation and oxidative stress by binding to their receptors (RAGEs). The leakage of toxic AGEs from the cells affects both hepatocytes (by worsening IR and elevating inflammatory marker levels) and hepatic stellate cells (by upregulating the expression levels of profibrotic markers). The levels of AGEs are higher in NASH patients when compared to healthy controls or the ones with simple steatosis [16]. These findings suggest that the inflammation caused by AGEs could be modulated. Whether IR precedes or follows the NAFLD is rather hard to tell, but the linkage between these two entities is undeniable. Recently, it has been shown that the eradication of HCV infection (with direct antiviral drugs) ameliorates IR, thus decreasing the chances for diabetes and cardiovascular disease to develop [17,18,19]. The improvement of IR in these cases occurs probably due to the reduction of chronic inflammation, by eliminating its cause. That new observations indirectly confirm the role of IR in the relationship between NAFLD and atherosclerotic disease. Given all that has been said, IR represents the most relevant pathogenetic feature that NAFLD (MAFLD) and MetS have in common, besides clinical features that these different entities share [20,21]. Also, the results reported by Masarone et al. (2017) demonstrated that the liver damage was associated with a progressively more severe clinical manifestation of IR, whereas histological features of NASH were observed in liver biopsies of patients with T2DM—more precisely in 98% of cases, suggesting that NASH may represent one of the early complications of T2DM due to its pathophysiological linkage with IR [22].

### 2.2. Lipotoxicity, Organelle Distress and Inflammasome Activation—The Road to NASH

**Figure 1:**
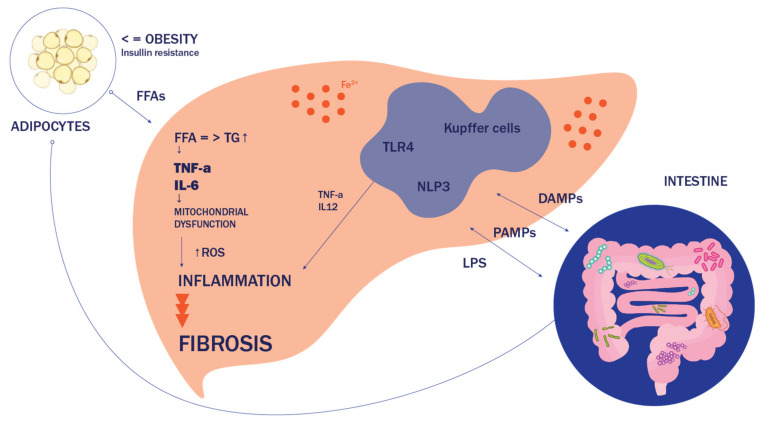
Molecular mechanisms in the pathophysiology of non-alcoholic fatty liver disease (NAFLD). Abbreviations: NAFLD, *non-alcoholic fatty liver disease*; FFAs, free fatty acids; TNF-α, tumor necrosis factor alpha; IL-6, interleukin 6; IL12, interleukin 12; TG, triglycerides; ROS, reactive oxygen species; LPS, lipopolysaccharide; PAMPs, pathogen-associated molecular patterns; DAMPs, damage-associated molecular patterns; TLR4, toll-like receptors; NLP3, NOD-, LRR-, and pyrin domain-containing protein 3.

Crosstalk at cellular and organ levels is necessary in the pathogenesis of NASH, specifically considering the relationship between adipose-liver and gut-liver axis (Figure 1). Once burdened with toxic lipid species, hepatocytes enter different kind of state which then triggers the cascade of processes leading to the development of NASH. Those toxic lipid molecules include, besides saturated fatty acids (like palmitate and stearate), diacylglycerols, ceramides, lysophosphatidyl-choline (LPC), and free cholesterol. At a molecular level, lipotoxicity leads to organelle stress and dysfunction, inflammasome activation and, eventually, to cell death and activation of inflammatory responses [23,24]. Given the major role of hepatic lipid equilibrium in the pathogenesis of NASH, it is only logical to assume that decreasing substrate influx to the liver, or enhancing discharge from the liver represent possible therapeutic strategies. This could be achieved, for example, by increasing FAO, inhibition of DNL, increasing fatty acid desaturation, or improving IR. Hepatocyte injury is followed by macrophage-mediated inflammation and hepatic stellate cells activation, comprising the key triad of NASH development. Lipotoxic species may activate apoptotic machinery in hepatocytes via either intrinsic or extrinsic (death receptor) pathways. Main molecular signaling occurs through c-Jun-N-terminal kinase (JNK) [25]. The JNK pathway is known to be stimulated by both oxidative stress (OS) and ER stress. At the organelle level, FFAs and their derivatives can induce mitochondrial over-function, followed by mitochondrial dysfunction, which results in increased levels of reactive oxygen species (ROS), the main culprit of oxidative stress [26]. Saturated FFAs, especially LPC derived from them, can also accumulate in the endoplasmic reticulum (ER) and trigger an ER stress, which stimulates the unfolded protein response (UPR), being an attempt of ER to re-establish its homeostasis. However, prolonged activation of the UPR leads to the activation of JNK or glycogen synthase kinase 3 (GSK3) and the induction of the transcription factor CCAAT/enhancer-binding homologous protein (CHOP), which all upregulate pro-death proteins, such asp53- upregulated modulator of apoptosis (PUMA) The extrinsic pathway includes tumor necrosis factor (TNF)–related apoptosis-inducing ligand (TRAIL)-induced cell death by transcriptional upregulation of TRAIL receptor 2 (TRAIL-R2) expression. Both pathways converge at a mitochondrial level when permeabilization of the mitochondrial outer membrane occurs, leading to the release of cytochrome c, activation of caspases, and apoptosis [27,28].

Inflammation represents a crucial aspect of NASH, and it can be originated from both extrahepatic sites, or within the liver, as a secondary response to lipotoxic attack [29]. The immunologic cascade starts with the recruitment of inflammasomes, intracellular multimeric protein complexes formed in order to activate caspase-1, an enzyme responsible for turning proinflammatory cytokines into their active forms, such as interleukin (IL)-1β and IL-18 [30]. The sensor proteins of these complexes are pattern recognition receptors (PPRs) that can be activated either by pathogen-associated molecular patterns (PAMPs) or damage-associated molecular patterns (DAMPs). Several types of PPRs are known, such as the NOD-like receptor proteins (NLRP), leucine-rich repeat (LRR)-containing proteins, and Toll-like receptors (TLRs) [31]. In NASH, PPRs ligands include lipopolysaccharide (LPS) derived from the gut or the signals from organelle distressed hepatocytes undergoing lipotoxicity or apoptosis. FFAs can directly activate some of the receptors, like TLR4. The effects of TLR4 are primarily driven by the NF-κB and mitogen-activated protein kinase (MAPK) cascades which, among other things, regulate inflammation [32]. In addition to inflamed hepatic tissue, the dysfunctional adipose tissue also releases proinflammatory cytokines such as TNF-α, IL-6, and IL-8, and chemokines—adipokines like monocyte chemotactic protein (MCP)-1. Other products of adipose tissue include hormones like leptin and adiponectin. It is known that adiponectin reduces IR and has anti-inflammatory and anti-steatotic effects, while leptin is identified as a profibrogenic adipokine [33]. The activation of Kupfer cells, liver resident macrophages, may be critical in NASH and it precedes the recruitment of other cells. Differentiation of Kupfer cells towards a proinflammatory (M1) phenotype is principally driven by PAMPs that in interaction with TLRs, induce the secretion of IL-1, IL-12, TNF-α, C-C motif chemokine- ligands 2 and 5 (CCL2 and CCL5) [34,35]. Chemotactic mediators like CCL2 and CCL5 are overexpressed in NASH, inducing other immune cells to infiltrate the liver, like CD4+ and CD8+ T-lymphocytes, B-lymphocytes, and natural killer cells (NKT). Being functionally active, recruited T- lymphocytes express markers CD44 and CD69, and increase the production of IFN-γ. Among CD4+ T-cells, it has been shown that the Th-17 family subset plays an important role in NAFL to NASH progression [36,37]. Further alterations of intercellular cross-talk in the inflamed liver could lead to the development of fibrosis, where hepatic stellate cells have the main role. The crucial step in fibrosis onset represents trans differentiation of previously dormant stellate cells into proliferative myofibroblasts—cells with strong profibrogenic transcriptional and secretory qualities, which further produce components of the extracellular matrix and form scar tissue [38,39].

### 2.3. Does the Answer Lie in the Gut?

Now well established, the term gut-liver axis represents the bidirectional connection between the gut and the liver, mainly through the biliary tract and portal and systemic circulation, i.e., enterohepatic circulation. Emerging evidence strongly suggests that gut microbiota may be the main intersection in this bidirectional communication [40]. Certain intestine hormones, like gastric inhibitory polypeptide (GIP) and glucagon-like peptide-1 (GLP-1), reduce hepatic glucose production and fat accumulation. Other hormones like fibroblast growth factor (FGF) 19 and FGF15 are reported to decrease hepatic lipogenesis. Being the main drivers of the intestinal hormone secretion, bile acids have an important place in NAFLD pathophysiology, because of their major role in lipid metabolism and their association with the establishment of the gut microbiota. They act on the nuclear farnesoid X receptor (FXR) and Takeda-G-protein-receptor-5 (TGR5) that are expressed on hepatic cells, among others, thus representing possible therapeutic targets [41,42].

During the last decade, extensive research has demonstrated that the microbiota can influence energy harvest and metabolism, therefore it is now considered a major environmental factor contributing to various states and diseases. So far, it has been shown both in animal models and in humans that the microbiota might undergo changes in composition and function towards distinctive phenotype which contributes to metabolic dysregulation [43,44,45]. More than a decade ago it was recognized that patients with NAFLD were characterized by dysbiosis—the state of an abnormal ratio of commensal and pathogenic bacterial species. Some species in humans were associated with NAFLD, such as Proteobacteria, Enterobacteria, Escherichia or Bacteroides, being higher in patients with NASH as compared to the matched healthy controls [46]. Also, it has been demonstrated that Firmicutes/Bacteroidetes ratio is disturbed in obese patients, as well as in those with NAFLD. Extensive recent research has shown that microbial signatures differ across different etiologies of chronic liver disease, as well as they are distinctive features of different stages of the disease, implicating a novel diagnostic biomarker and a very possible therapeutic target [47,48].

Several mechanisms may underlie the role of microbiota in NAFLD development and evolution, and they will be discussed in the following section.

#### 2.3.1. Increased Gut Permeability

The intestinal barrier has a crucial role in maintaining the normal environment of the intestinal tract, preventing harmful substances (toxins, microbes, and bacterial metablites) from escaping the lumen while obtaining vectorial transport of nutrients, water, and waste products [49]. The defensive strategies include mechanical ones such as the presence of tight junctions (TJs) between the adjacent epithelial cells, or secretion of mucus, antimicrobial peptides (APs), and different types of cytokines. Dysbiosis in a high-fat diet (HFD) causes an impairment of the intestinal barrier, known as the leaky gut syndrome, mainly through changes in expression and distribution of TJs, and a decrease in the secretion of APs. In that way, the translocation of bacteria or bacterial products, such as LPS, into the portal circulation is enabled. Although increased intestinal permeability is not the main cause of NASH, it warrants an inducement of a strong liver inflammatory response to microbial antigens, which represents the major step in NAFL progression to NASH [36,50,51].

#### 2.3.2. Disruption of Inflammatory Response and LPS Endotoxemia

The components of the immune system (especially the innate immune response) are crucial for maintaining the proper tolerance to the gut microbiota and for controlling bacterial growth and pathogenicity. The secretion of IgA by B cells as a part of the adaptive immune system also represents an important mechanism in the control of the gut microbiota. The presence of dysbiosis can cause modifications in this cross-talk between the immune system and the gut [52]. Among the innate system response, PPRs are responsible for sensing bacterial components and products. When bacterial translocation occurs, these components trigger an immunologic cascade. It has been observed both in animal and human models that HFD alters the gut microbiota in such a way that favors an increase in systemic levels of LPS, the major outer membrane component of Gram-negative bacteria. With previously increased intestinal permeability, LPS can enter into the portal circulation causing metabolic endotoxemia i.e., low-grade inflammation [53]. This immunologic cascade started by the LPS is considered to have a pivotal role in NASH development. Higher levels of LPS, as well as the LPS-binding protein (LBP), occurred in patients with NASH when compared to the patients with simple steatosis or healthy controls [54,55,56]. The LPS is recognized by TLRs (especially TLR4). The stimulation of TLR4 triggers several intracellular signaling pathways, inducing the synthesis of proinflammatory cytokines, mostly TNFα, which further causes inflammation, oxidative stress, and the promotion of IR. Kupffer cells, being the liver cells with the highest expression of TLR4 are the ones that respond to LPS by producing cytokines and ROS. Stellate cells are also activated in this interaction thus inducing hepatic fibrogenesis [41,57]. Besides the activation of the membranous TLR4, LPS can also cause liver injury through a mechanism mediated by the intracellular components, inflammasomes (especially NLRP3), which is shown to be linked with the progression of NASH [33,58,59]. Other receptors involved in the microbiota-related mechanisms of the NASH development are TLR2, TLR5, and TLR9, which recognize peptidoglycans, flagellins, and bacterial DNA, respectively [60].

#### 2.3.3. Microbiota-Related Alterations of Host Metabolism

The large impact on host metabolism by the gut microbiota is primarily defined through its capacity to process indigestible polysaccharides to short-chain fatty acids (SCFAs), mainly acetate, propionate, and butyrate, thus producing extra energy for the host. It has been demonstrated that these SCFAs have an overall positive effect on lipid and glucose metabolism, by their binding to the specific G-protein–coupled receptors (GPRs) 41 and GPR43, or through the activation of PPARγ. The expression of GPRs has been identified in the adipose tissue, liver, and intestine [26,34]. Also, the important impact of microbiota on bile acids metabolism and vice versa has already been elucidated. Colonic bacteria can metabolize deconjugated bile acids into secondary bile acids and facilitate their excretion through feces. Microbial processing of bile acids increases the diversity of the bile acid pool and therefore influences lipid metabolism through the interaction of bile acids with FXR or TGR5, acting as their agonists or antagonists [61,62]. One of the other abilities of intestinal bacteria is the inhibition of the synthesis of fasting-induced adipocyte factor, also known as angiopoietin-related protein 4. This factor acts as an inhibitor of the lipoprotein lipase (LPL), thus increasing the discharge of FFAs from VLDL particles into the liver [36]. Other microbial products including trimethylamine *N*-oxide (TMAO) or ethanol, may be involved in the regulation of insulin sensitivity, metabolic inflammation, and can even affect the immune system, contributing to the development of NAFL and progression to NASH. It has been shown that systemic ethanol levels are higher in NASH patients, revisiting a hypothesis that ethanol-producing microbial strains might be involved in the pathogenesis of NASH [49,63].

As we are still trying to elucidate the complex mechanisms of host-microbial interactions, future studies will have to show more precise results. A key question is whether a disease-promoting microbiota can be modified either by a dietary approach, by the use of pre/probiotics, or even fecal transplantation in patients with NAFLD/NASH [64,65].

## 3. Innovative Therapeutic Strategies in NAFLD

**Figure 2:**
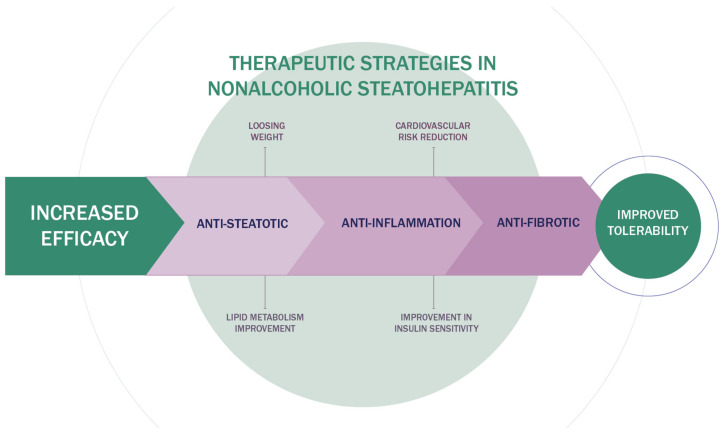
Therapeutic algorithm for NAFLD treatment.

Given the complex pathogenesis of NASH, therapeutic options are focused on hepatic steatosis, inflammation, and fibrosis. The ideal combination of drugs should have beneficial effects on the liver, weight loss, cardiovascular system, glucoregulation, and lipid regulation [66,67]. Therefore, the therapeutic approach to NASH should be multidisciplinary. A cardiologist, hepatologist, endocrinologist, and nutritionist should participate in the treatment of the NASH patients. The new therapeutic algorithm for NAFLD is shown in Figure 2.

### 3.1. Lifestyle Modification

Lifestyle modification using diet and exercise to achieve weight loss remains a first-line intervention in patients with NAFLD [68]. Mediterranean diet (MD) may improve NAFLD by modulating the presence of visceral obesity, insulin resistance, dyslipidemia, and chronic inflammation, all features of metabolic syndrome. MD is rich in macronutrients that have a beneficial effect on glucidic and lipidic metabolism and, consequently, on fatty liver disease. The MD is high in mono-unsaturated fatty acids (MUFAs), poly-unsaturated fatty acids (PUFAs), and fibers, and low in refined sugars and fructose. MUFAs reduce risk factors for metabolic syndrome. PUFAs, particularly omega-3 fatty acids, have been shown to be beneficial in preventing cardiovascular events [69]. Data suggest that omega-3 polyunsaturated fatty acids (n-3 PUFA) supplementation may be effective in the early stages of NAFLD, but not in patients with more severe NAFLD or NASH [70]. A sedentary lifestyle is a risk factor for the development of obesity, T2DM, NAFLD, and metabolic syndrome. The results of many studies emphasize that physical activity reduces the body weight (at best > 10%) and thus improves liver histology and reduces fibrosis in NAFLD patients. Physical activity and specially structured exercises are the main treatments for NAFLD patients, independent of weight loss. Both aerobic and resistance training effectively reduce hepatic steatosis and reduce the NAFLD-associated cardiovascular risk [71].

### 3.2. Antioxidant

Vitamin E is well known as an antioxidant and is expected for the treatment of NASH. In PIVENS trial, vitamin E (800 mg/day) was superior to placebo for the improvements of NASH histology in adult NASH patients without diabetes. However, fibrosis improvement was not confirmed. Due to its adverse effects profile and lack of significant improvement in hepatic fibrosis, current guidelines recommend that vitamin E-based treatment be restricted to biopsy-proven nondiabetic patients with NASH only [72].

### 3.3. Farnesoid X Receptor (FXR) Agonists

Obeticholic acid (OCA), a ligand of FXR, is a synthetic derivate of natural bile acid chenodeoxycholic acid. Drugs-activating FXR has shown effects in cholestatic liver disease. OCA is the most advanced FXR agonist in the treatment of NASH [41]. In clinical trials, obeticholic acid has demonstrated efficacy in the resolution of NASH without the worsening of fibrosis [73,74].

Due to major adverse effects related to obeticholic acid such as pruritus and increased low-density lipoprotein (LDL) cholesterol, non-bile acid FXR agonists have been developed [75]. Tropifexor showed a decrease in steatosis and alanine aminotransferase (ALT) and gamma-glutamyl transferase (GGT) serum levels [76]. Cilofexor and nidufexor are new FXR agonists whose effects on NASH are being investigated [77,78,79].

### 3.4. Peroxisome Proliferator-Activated Receptor (PPARs) Agonists

PPAR-α, PPAR-δ, and PPAR-γ have anti-inflammatory effects and have a role in lipid and glucose metabolism [80]. The PPAR-γ agonist, pioglitazone, is the most used one [81,82,83]. The American Association for the Study of Liver Diseases (AASLD) and European Association for the Study of Liver (EASL) guidelines recommend pioglitazone for use in patients with biopsy-proven NASH [2,5]. However, PPAR exhibit many side effects such as weight gain, fluid retention, and the increased risk of bone fracture. To avoid side effects, clinical trials with dual PPAR agonists are ongoing: elafibranor is a PPAR-α and PPAR-δ agonist, saroglitazar is a PPAR-α and PPAR-γ agonist and lanifibranor is a pan-PPAR agonist [84,85,86].

### 3.5. Glucagon-Like Peptide-1 (GLP-1) Agonists

As previously mentioned, GLP-1 is a hormone which stimulates glucose-dependent insulin secretion and inhibits glucagon secretion, thus regulating plasma glucose levels. Also, GLP-1 activates GLP-1 receptors in the hypothalamus, increases satiety and reduces gastric emptying time, and induces weight loss [87]. In the clinical NAFLD study, in patients with uncontrolled T2DM, GLP-1 agonist, liraglutide leads to weight loss and liver fat reduction [88].

The new generation of GLP-1 agonist semaglutide is currently under investigation for the use in obesity and NASH [89]. In a new study, the treatment with subcutaneous semaglutide resulted in a significantly higher percentage of patients with NASH resolution than placebo. However, the trial did not show a significant improvement in the fibrosis stage [90].

### 3.6. Sodium/Glucose Transport Protein 2 (SGLT2) Inhibitors

SGLT2 inhibitors are a class of antidiabetic agents which induce moderate weight loss and reduce body fat, mainly by increasing urinary glucose excretion. These agents may exert cardiovascular and renal protection in T2DM patients [91]. The SGLT2 inhibitor, dapagliflozin decreases hepatic fat content but has no major effects on tissue-specific insulin sensitivity [92,93]. Empagliflozin, another SGLT2 inhibitor, reduces hepatic fat in patients with T2D with excellent glycemic control, decreases circulating uric acid, and raises adiponectin levels despite unchanged insulin sensitivity. The drug can, therefore, be considered in the early treatment of nonalcoholic fatty liver disease in T2D [94].

### 3.7. Probiotics and Synbiotics

Meta-analysis involving 782 patients with NAFLD showed evidence for the benefit of probiotics and synbiotics supplementation for liver steatosis, liver enzymes, lipid profiles, and liver stiffness [95].

Results of VSL3 formulation study (including probiotic bacterial strains *S. thermophiles*, *Bifidobacterium breve*, *Bifidobacterium longum*, *Bifidobacterium infants*, *Lactobacillus casei*, *Lactobacillus plantarum*, *Lactobacillus acidophilus*, and *L. bulgaricus*) in children with NAFLD showed significant improvement of NAFLD and weight loss [96].

The conclusion of a randomized clinical trial was that multi-strain probiotic (14 probiotic bacteria genera Bifidobacterium, Lactobacillus, Lactococcus, Propionibacterium) reduces the fatty liver index, cytokines, and aminotransferase levels in NAFLD patients [97].

The result of a randomized, double-blind, placebo-controlled clinical trial was that synbiotic supplementation with seven probiotic strains (*Lactobacillus casei*, *L. bulgaricus*, *Lactobacillus rhamnosus*, *Lactobacillus acidophilus*, *Bifidobacterium breve*, *B. longum*, and *S. thermophilus*) and fructo-oligosaccharides with healthy lifestyle modifications, was very beneficial to NAFLD patients, [98].

There has been a growing interest in the use of fecal microbiota for the treatment of patients with NASH and NAFLD. Based on the results of ongoing clinical trials we can expect a potential therapeutic role of fecal microbiota transplantation for the treatment of NASH and NAFLD [99,100].

### 3.8. Lipogenesis Inhibitors

Cholesterol and triglyceride are the key toxic lipid mediators of liver injury.

Stearoyl-CoA desaturase-1 (SCD-1) converts saturated FAs to monounsaturated FAs [101]. The deficiency or inhibition of SCD-1 leads to a reduction in liver steatosis and improvement in insulin sensitivity. Obese patients with NASH have increased SCD-1 activity [102]. Aramchol is l SCD-1 inhibitor in the liver. A placebo-controlled clinical trial in NAFLD patients reported a reduction in hepatic fat content and fibrosis after the administration of aramchol [103].

Acetyl-CoA carboxylase (ACC) converts acetyl-CoA to malonyl-CoA [104]. Inhibition of ACC reduces hepatocellular malonyl-CoA levels and, in that way, decreases hepatic lipid synthesis while simultaneously increasing FAO. A clinical trial with liver-targeted ACC inhibitor GS-0976 (Firsocostat) showed that patients receiving the higher dose of GS-0976 (Firsocostat) demonstrated a significant reduction in liver fat content [105].

Statins are not currently recommended for NASH treatment, because they do not have favorable effect on liver histology but only reduce cardiovascular disease risk [106].

### 3.9. Thyroid Hormone Receptor Beta (TRβ) Agonists

TRβ agonists stimulate fatty acid β-oxidation and oxidative phosphorylation in the liver [107]. Numerous studies have shown that patients with hypothyroidism and subclinical hypothyroidism have an increased risk for NAFLD. In a phase 2b trial, Harrison et al. have recently demonstrated that resmetirom significantly reduces liver fat content. Resmetirom is now being assessed in a large, phase 3 MAESTRO-NASH clinical trial to test its efficacy and safety in patients with NASH [108]. Resmetirom has no effect on liver fibrosis, but it can be assumed that it may be more effective in earlier-stage NASH when the fibrosis is still reversible. Since the drug is not expensive it can be used in poor countries with a large number of patients with NAFLD. The drug has a few serious side effects, primarily atrial arrhythmias, and osteoporosis, and it causes small changes in FT4 levels. Resmetirom could be combined with other drugs with other mechanisms of NAFLD [109].

### 3.10. CCL Receptor Type 2 (CCR2) and Type 5 (CCR5) Antagonists

CCR2 plays a role in the infiltration of proinflammatory mediators at the site of liver injury, and CCR5 in the proliferation of collagen-producing cells. Cenicriviroc (CVC) is a novel, orally administered, potent chemokine 2 and 5 receptor antagonist currently being developed for the treatment of liver fibrosis in adults with NASH. The final result of the phase 2b CENTAUR trial is that the antifibrotic effect of CVC is maintained in the second year of treatment of the patients with NASH and liver fibrosis. The antifibrotic effect was greater in advanced fibrosis. The drug is well tolerated. [110]. The AURORA study will be conducted to confirm the efficacy and safety of CVC for the treatment of liver fibrosis in adult subjects with NASH. However, there are concerns about the duration of the antifibrotic action of CVC and the different effects on NASH and fibrosis. Due to the above-mentioned, the drug will probably not be approved soon [111].

### 3.11. Fibroblast Growth Factor-19 (FGF19) Analogue

FGF19 plays a central role in regulating bile acids, carbohydrates, and energy metabolism T. Bile acids are an important risk factor for chronic liver diseases and play a central part in the pathogenesis of NAFLD. Aldafermin is an engineered, analogue of the human gut hormone, FGF19. In phase 2 trial of patients with NASH, aldafermin reduced liver fat and produced a trend toward fibrosis improvement. Fibrosis improvement with no worsening of NASH was observed in 38% of patients given aldafermin vs. 18% of patients given the placebo. There were few adverse events and most of them were of mild or moderate severity [112]. However, the limitation of this study is the small number of patients. The approval of the use aldafermin in the treatment of NAFLD requires larger studies.

In phase 2 clinical study, administration of aldafermin, in NASH patients was associated with an increase of the genus Veillonella in stool samples, which was also associated with an improvement in the serum bile acid profile and reduced liver fat content [113]. The results of this study indicate the possible importance of the gut microbiota in both pathogenesis and NAFLD therapy.

## 4. Combination Therapy of NASH

Since NASH is a multifactorial disease involving different contributing mechanisms, combination therapy is likely to show additional benefits when compared with monotherapy.

TANDEM study will evaluate the combination of tropifexor, a farnesoid X receptor agonist, and cenicriviroc, a chemokine receptor types 2/5 antagonist. Therapy is targeted at inflammation and fibrosis in patients with NASH [114]. Clinical efficacy superior to most other treatment options was shown by FXR agonists such as obeticholic acid (OCA) as they improved various metabolic features including liver steatosis, liver inflammation, and fibrosis [115].

Since there is a frequent association between diabetes mellitus type 2 and NASH, the combination of anti-diabetic drugs with anti-NASH drugs may help improve both diseases.

The combination of pioglitazone and vitamin E resulted in a numerically greater response in the resolution of NASH and improvement of fibrosis when compared with the use of pioglitazone alone [116].

The combined therapy, with antidiabetics and weight-loss drugs, such as GLP-1 agonist and SGLT2 inhibitors, has a great significance in the therapy of NASH [117].

Another combination of therapies is to reduce the side effects of the drugs. For example, FXR agonists increase LDL cholesterol and the combination with a statin may decrease this side effect. This was tested in the study with OCA and atorvastatin [118]. Also, due to hypertriglyceridaemia during the therapy with ACC inhibitor, the combination with fenofibrate may decrease this side effect. The results of the study using the combinedtherapy of fenofibrate and firsocostat in patients with NASH showed an increase in triglycerides but also an improvement in hepatic steatosis [119].

Pentoxifylline (PTX) is a phosphodiesterase inhibitor. It has antioxidant and anti-inflammatory actions. A randomized placebo-controlled trial noted that PTX markedly reduced steatosis, lobular inflammation, and liver fibrosis [120]. Metformin (MET) exerts its effect on glucose homeostasis via adenosine monophosphate-activated protein kinase. Current information indicates that metformin improves liver function, HOMA-IR, and BMI to some extent, but not histological response in NAFLD patients [121]. Results of a prospective trial showed that combination therapy PTX and MET is a new potential benefit in improving liver function and decreasing cardiovascular risk [122].

In the experimental model, Liu and co-authors (2021) identified HMG-CoA reductase degradation protein (Hrd1) as a novel E3 ligase for ATP citrate lyase (Acly). Negative regulation of Acly by Hrd1 reduces acetyl-CoA levels and inhibits lipid synthesis in hepatocytes.Increased hepatic Hrd1 also improves carbohydrate metabolism by affecting some regulators. These results could represent a foundation of the new NAFLD therapeutic approach [123,124]. An overview of innovative therapeutic strategies in the treatment of NAFLD is shown in Table 1.

## 5. Conclusions

As the most common chronic liver disease in the world, NAFLD represents a great global burden, with mortality and morbidity not only related to the liver but also including several extra-hepatic organ systems. In addition to previously established linkages among different pathophysiological processes involved in NAFLD development, recent research efforts are constantly presenting new evidence strongly suggesting that gut microbiota may be the key link, having a pivotal role in the pathogenesis of NAFLD and its progression to NASH and cirrhosis. With new data rapidly emerging, the new therapeutic approaches are simultaneously being developed, focusing on and considering all the facts mentioned above. Assessment of NAFLD demands a multidisciplinary approach in order to early recognize and treat this multisystemic disease, thus reducing the effects of this serious public health problem.

## Figures and Tables

**Table 1:** Innovative therapeutic strategies in NAFLD.

Pathway	Mechanism of Action	Drug
Metabolism	Farnesoid X receptor(FXR) agonists	Obeticholic acid (OCA)
		Tropifexor
		Cilofexor
		Nidufexor
	Peroxisome proliferator-activated receptor (PPARs) agonists	Pioglitazone
		Elafibranor
		Saroglitazar
		Lanifibranor
	Acetyl-CoA carboxylase inhibition	Aramchol
		Firsocostat
	Glucagon-like peptide-1(GLP-1) agonists	Liraglutide
		Semaglutide
	Thyroid hormone receptor beta (TRβ) agonists	Resmetirom
	Fibroblast growth factor-19 (FGF19) analogue	Aldafermin
	Sodium/glucose transport protein 2 (SGLT2) inhibitors	Dapagliflozin
		Empagliflozin
Inflammation	CCL receptor type 2 (CCR2) and type 5 (CCR5) antagonists	Cenicriviroc
Gut- liver axis	Probiotics, symbiotics	VSL3 Multi-strain probiotic (14 probiotic bacteria genera Bifidobacterium, Lactobacillus, Lactococcus, Propionibacterium) Synbiotic (7 probiotic strains *Lactobacillus casei*, *L. bulgaricus*, *Lactobacillus rhamnosus*, *Lactobacillus acidophilus*, *Bifidobacterium breve*, *B. longum*, and *S. thermophilus*)
Cell death	Antioxidant	Vitamin E

## Data Availability

The literature data used to support the findings of this study are deposited in the DOI repository.
